# Effect of 6-Methoxybenzoxazolinone on the Cecal Microbiota of Adult Male Brandt’s Vole

**DOI:** 10.3389/fmicb.2022.847073

**Published:** 2022-03-29

**Authors:** Xin Dai, Lin Chen, Mengyue Liu, Ying Liu, Siqi Jiang, Tingting Xu, Aiqin Wang, Shengmei Yang, Wanhong Wei

**Affiliations:** ^1^College of Bioscience and Biotechnology, Yangzhou University, Yangzhou, China; ^2^Jiangsu Co-Innovation Center for Prevention and Control of Important Animal Infectious Diseases and Zoonoses, Yangzhou University, Yangzhou, China

**Keywords:** 6-MBOA, cecal microbiota, SCFAs, KEGG pathway analysis, Brandt’s vole

## Abstract

The anti-microbial effects of plant secondary metabolite (PSM) 6-methoxybenzoxazolinone (6-MBOA) have been overlooked. This study investigated the effect of 6-MBOA on the cecal microbiota of adult male Brandt’s voles (*Lasiopodomys brandtii*), to evaluate its effect on the physiology of mammalian herbivores. The growth of voles was inhibited by 6-MBOA. A low dose of 6-MBOA enhanced the observed species, as well as the Chao1 and abundance-based coverage estimator (ACE) indices and introduced changes in the structure of cecal microbiota. The abundance of the phylum Tenericutes, classes Mollicutes and Negativicutes, order Selenomonadales, families *Ruminococcaceae* and *Veillonellaceae*, genera *Quinella*, *Caproiciproducens*, *Anaerofilum*, *Harryflintia*, and unidentified *Spirochaetaceae* in the cecal microbiota was enhanced upon administration of a low dose of 6-MBOA, which also inhibited glucose metabolism and protein digestion and absorption in the cecal microbiota. 6-MBOA treatment also stimulated butyrate production and dose-dependently enhanced the metabolism of xenobiotics in the cecal microbiome. Our findings indicate that 6-MBOA can affect Brandt’s voles by inducing changes in the abundance of cecal bacteria, thereby, altering the contents of short-chain fatty acids (SCFAs) and pathway intermediates, ultimately inhibiting the growth of voles. Our research suggests that 6-MBOA could potentially act as a digestion-inhibiting PSM in the interaction between mammalian herbivores and plants.

## Introduction

Plant secondary metabolites (PSMs) are chemical compounds that play an important role in defense against herbivores, with likely effects on herbivore physiology and behavior (Freeland and Janzen, 1974; Hughes, 1988). Conversely, herbivores have evolved several strategies in response to PSMs, such as gut microbial detoxification (Jones and Megarrity, 1986; Dearing et al., 2005; Sundset et al., 2010; Johnson et al., 2018). Gastrointestinal microbes play a significant role in host metabolism and are essential modulators of body homeostasis and health (De Filippo et al., 2010; Sommer and Bäckhed, 2016). Gut microbial metabolites, such as short-chain fatty acids (SCFAs), have been shown to mediate the effects of gut microbiota on their hosts (Koh et al., 2016). Therefore, changes induced in the gut microbiota in response to PSM ingestion would have profound effects on herbivores, such as nutrient digestion and absorption and materials metabolism, not exclusively due to gut detoxification. However, the response of herbivore gut microbiota to PSMs has not been thoroughly studied, and the role of the gut microbiota in the interaction between plants and herbivore is thus not yet fully understood.

The PSM 6-methoxybenzoxazolinone (6-MBOA) is mainly produced during the early growth stages of plants belonging to the family Gramineae (Argandoña et al., 1981; Niemeyer, 1988; Acharya et al., 2021). Although 6-MBOA is mainly known to stimulate the reproduction of certain animals (Dai et al., 2016) such as rodents (Berger et al., 1981; Alibhai, 1986; Anderson et al., 1988; Nelson and Shiber, 1990; Martin et al., 2008; Dai et al., 2016) and rabbits (Rodríguez-De Lara et al., 2007), it has diverse effects and acts as a defense compound against insect feeding and digestion (Klun and Brindley, 1966; Campos et al., 1988; Houseman et al., 1992; Dowd and Vega, 1996; Maag et al., 2014), in addition to restraining the growth of microbes such as *Escherichia coli*, *Proteus rulgaris*, *Cephalosporium gramineum*, *Fusarium oxysporum*, *Coprinus comatus*, *Rhizoctonia solani*, and *Pythium* species (Wang et al., 2001; Wang and Ng, 2002; Martyniuk et al., 2006; Acharya et al., 2021). This metabolite can also alter root-associated microbiota in maize (Hu et al., 2018). Additionally, 6-MBOA can influence animal and human metabolism such as the ameliorating glucose tolerance in diabetic rats (Hameed et al., 2019) and obesity in humans (Fomsgaard et al., 2011). However, to date, little has been known about the effect of 6-MBOA on the gut microbiota in mammalian herbivores and its potential effects on the physiology of mammalian herbivores mediated by the gut microbiota.

Brandt’s vole (*Lasiopodomys brandtii*) is a small and seasonally reproductive mammal widely distributed in the grasslands of Inner Mongolia, China (Xie et al., 1994; Shi et al., 1999; Wan et al., 2002a). This species feeds on a wide variety of herbal plants, favoring *Leymus chinensis, Medicago varia, Stipa krylovii*, and *Saussurea runcinata* (Wang et al., 1992; Li et al., 2019). It has been reported that gut microbiota composition in Brandt’s vole can be affected by multi-factors, such as temperature, housing density, stress, diet, age, and gender (Bo et al., 2019; Li et al., 2020; Liu J. et al., 2020, 2021; Xu and Zhang, 2021). Previously, we detected 6-MBOA in the seedlings of *L. chinensis*, which is dominant in the grasslands of Inner Mongolia (Wang et al., 1992; Li et al., 2019), with the highest concentration of 6-MBOA exceeding the 100 mg/kg during seedling germination (Dai et al., 2014). Therefore, Brandt’s vole represents an ideal model for studying the effect of 6-MBOA on the structure and function of the gut microbiota in mammalian herbivores.

In this study, we investigated the effects of different doses of 6-MBOA on the body weight growth, cecal SCFAs, alpha and beta diversities, and functions of the cecal microbiota of adult male Brandt’s voles. These results provide new insights into the effect of 6-MBOA on the physiology of mammalian herbivores and the mechanisms of their responses to 6-MBOA. We illustrate the potential role of 6-MBOA in plant-mammalian herbivore interactions and contribute to the theory of coevolution between plants and animals.

## Materials and Methods

### Animals and Treatments

Our study was conducted at the College of Bioscience and Biotechnology, Yangzhou University, China. Brandt’s voles captured from the grasslands of Inner Mongolia were bred in an animal house at Yangzhou University, Jiangsu Province, China. The following were the environmental conditions employed: temperature, 22 ± 1°C; relative humidity, 50 ± 5%; photoperiod, 12 h light/12 h dark (light period: 06:00–18:00). Newborn voles were weaned at 21 days and individually housed in polypropylene cages until they were 90-days old. Because both sex-hormone levels and age affect the gut microbiota community of Brandt’s vole (Xu and Zhang, 2021), and female Brandt’s voles have complex variations in sex-hormone levels during the sexual cycle, adult male voles were selected as experimental voles in this study. At 90 days, 24 adult male voles were chosen from different families to guarantee that these voles were neither siblings nor half-siblings. The voles were then randomly assigned to one of three groups (control, low 6-MBOA dose, and high 6-MBOA dose), with eight individuals per group. All voles were provided *ad libitum* access to filtered tap water and standard rodent chow before and throughout the experimental period. The nutrient contents of the rodent chow (Yizheng Animal Biotechnology Co., Ltd., Yangzhou, China) were as follows: crude protein, ≥ 18%; crude fat, ≥ 4%; crude fiber, ≥ 5%; ash, ≤ 8%; calcium, 1.0–1.8%; phosphorus, 0.6–1.2%. All procedures were approved by the Animal Care and Use Committee of the Faculty of Veterinary Medicine of Yangzhou University (No. NSFC2020-SKXY-6).

A stock solution (200 μg/mL) of 6-MBOA (95% purity; Shanghai ZZBIO Co., Ltd., China) was prepared by dissolving 6-MBOA in distilled water and storing it at 4°C. On the day of gavage (intragastric administration), the stock solution was brought to 22°C and diluted twofold with distilled water. The exact volume of 6-MBOA administered to each vole was calculated according to the body weight of the vole to ensure that each vole in the low or high 6-MBOA dose groups received 1 or 2 mg/kg 6-MBOA per day, respectively. According to the average concentration of 6-MBOA (≥ 50 mg/kg 6-MBOA) in the *L. chinensis* seedling (Dai et al., 2014) and field consumption by Brandt’s vole (Wan et al., 2002b), a wild vole weighing 50 g can obtain 100 μg of 6-MBOA daily in the early spring, corresponding to the high dosage of 6-MBOA treatment (2 mg/kg 6-MBOA per day) in the present study, provided it consumes only 2 g of *L. chinensis* seedling. Therefore, the selection of the dosages of 6-MBOA in the present study was rational and should depend on the 6-MBOA ingestion by wild male voles. Each vole in the low or high 6-MBOA dose group was administered 100 μg/mL 6-MBOA solution or 200 μg/mL 6-MBOA solution, respectively. The control group was administered an equivalent volume of distilled water. Gavage was conducted every 2 days at approximately 09:00 a.m. for 15 days. Voles were weighed every 2 days from days 1 to 16. The food consumption of the voles was measured every 2 days from days 1 to 16 as the weight of chow given on the previous day minus the weight of remaining chow on the next day. On day 16, all animals were weighed and decapitated after anesthetization with ether. The cecal content was collected and immediately refrigerated in sterile tubes at −70°C for the analysis of SCFA concentration and 16S rRNA gene sequencing. The growth rate was calculated as the body weight difference between day 16 and 1 divided by the weight on day 1. Relative food consumption was calculated as the average food consumption divided by the weight of voles on the same day.

### DNA Extraction and 16S rRNA Gene Sequencing

To save sequencing costs, samples of cecal content were taken from only six voles randomly selected out of eight voles in each dose group for DNA extraction and 16S rRNA gene sequencing of the cecal microbiota by a Chinese company (Novogene Co., Ltd., Beijing, China). All procedures were performed according to the methods established by Novogene. Total genomic DNA from the samples was extracted using the CTAB/SDS method. DNA concentration and purity were evaluated using a 1% agarose gel. DNA was diluted to 1 ng/μL using sterile water. Using 10 ng of microbial genomic DNA as the template, universal primers (515F: 5′-GTGCCAGCMGCCGCGGTAA-3′; 806R: 5′-GGACTACHVGGGTWTCTAAT-3′) were used to amplify the V4 hypervariable region of the prokaryotic 16S rRNA gene. All PCRs were conducted in 30 μL reaction volume composed of 15 μL of Phusion ^®^ High-Fidelity PCR Master Mix (New England Biolabs). PCR products were mixed with 1 × loading buffer containing SYBR Green (Thermo Scientific) and electrophoresed on 2% agarose gel for confirmation. Then, the PCR products were purified with the GeneJET™ Gel Extraction Kit (Thermo Fisher Scientific). Sequencing libraries were generated using the Ion Plus Fragment Library Kit 48 rxns (Thermo Fisher Scientific), as per the manufacturer’s instructions. The quality of the prepared library was assessed on a Qubit 2.0 Fluorometer (Thermo Fisher Scientific). Finally, the library was sequenced on an Ion S5TM XL platform. Filtered high-quality classifiable 16S rRNA gene sequences (252–253 bp single-end reads) were generated.

### Bioinformatic Analysis

Single-end reads were assigned to each sample referring to their barcodes and trimmed by cutting off the barcodes and primer sequence using the Cutadapt (V1.9.1) (Martin, 2011). The reads were compared with the Silva database (Quast et al., 2013) using the UCHIME algorithm (Edgar et al., 2011) to remove the chimera sequences (Haas et al., 2011) and finally obtain the clean reads. Sequence analysis was performed by Uparse software (Uparse v7.0.1001) (Edgar, 2013). A minimum identity of 97% was used as the threshold for any sequence pair to identify different bacterial operational taxonomic units (OTUs). Taxonomic information of OTU sequences was annotated using the Silva132 Database^1^ (Quast et al., 2013) with the Mothur algorithm. The SSUrRNA databases of Silva132 were used for species annotation analysis (threshold: 0.8–1) (Haas et al., 2011; Edgar, 2013). The sequence data are available at the NIH Sequence Read Archive^2^ with the Bioproject ID PRJNA768324. The OTU abundance information was normalized using a standard sequence number corresponding to the sample with the least sequences. Subsequent analyses of the alpha and beta diversity were performed based on the output normalized data. The complexity of species diversity of a sample was analyzed *via* alpha diversity based on five indices, including observed species (OBSP), Chao1, abundance-based coverage estimator (ACE), Shannon, and Simpson. All indices were calculated using Quantitative Insights Into Microbial Ecology (QIIME, Version1.7.0). To identify significant biomarkers among the three groups, a linear discriminant analysis (LDA) effect size (LEfSe) analysis was performed using the online LEfSe program on the Huttenhower Lab server^3^ (Liu T. H. et al., 2020) and a default setting of 2 for the LDA score.

### Kyoto Encyclopedia of Genes and Genomes Pathway Prediction

The biological functions of the cecal bacterial community were predicted and annotated using the Kyoto Encyclopedia of Genes and Genomes (KEGG) pathway database along with the PICRUSt program on the Huttenhower Lab server (see text footnote 3) based on the result of the 16S rRNA gene sequencing (Chen et al., 2020).

### Short-Chain Fatty Acids Measurement

The levels of six SCFAs, i.e., acetate, propionate, butyrate, isobutyrate, valerate, and caproate in the cecum were measured by high-performance gas chromatography (GC; Agilent 7890A; Agilent Technologies, Germany) coupled with a GC autosampler and an FID system, using a modified method (Zhang et al., 2018). Frozen cecal contents were first thawed on ice, and then 0.5 mL of the thawed cecal content samples was weighed. One milliliter of distilled water was added to each sample for dilution. Standards were prepared by mixing 990 μL of distilled water, 5 μL of acetate, and 1 μL of each of the other SCFAs. Separations of the SCFAs were performed at 250°C on a 30 m × 0.25 mm × 0.25 μm DB-WAX column (Agilent Technologies) using 99.998% hydrogen as the carrier gas at a flow rate of 1.0 mL/min. Injections were performed in splitless mode at 230°C, and 0.5 μL was used for each injection. The oven temperature was set at 60°C for 1 min, increasing to 200°C at 5°C/min, and then further increasing from 200 to 230°C at 10°C/min. An Agilent chemstation was used to calculate the peak area of each SCFA in the standards as well as in the samples. The total running time for each sample was 32 min. The volume of each SCFA in the 0.5 μL samples was calculated as the ratio of the peak area of each SCFA in the sample to that in the standards, multiplied by the volume of each SCFA in the 0.5 μL standards. The total volume of each SCFA in each 0.5 mL cecal content sample was calculated as the volume of each SCFA in the 0.5 μL samples multiplied by the ratio of 1.5 mL to 0.5 μL. Finally, the concentration of each SCFA in the cecal content sample was calculated as the volume of each SCFA in each 1.5 mL sample divided by the weight of the 0.5 mL sample.

### Statistical Analysis

The standard non-parametric Kruskal–Wallis test was used to determine the differences in OTU biomarkers, alpha diversity indices, SCFA concentrations, and pathway enrichment among the three groups. The differences in total tags, taxon tags, and OTU numbers among the three groups were compared, and the effects of 6-MBOA on the growth rate and relative food consumption were determined using one-way analysis of variance (ANOVA) followed by Tukey’s *post-hoc* test because these data showed homogeneity and normality. Significant differences in the beta diversity of the cecal microbial community were evaluated by permutational multivariate analysis of variance (PERMANOVA) with Bray–Curtis distance matrices with nested adonis function in R (version 4.0.4) with the “vegan” package. A principal coordinates analysis (PCoA) based on Bray–Curtis dissimilarity was conducted to depict the dissimilarities in the profiles of the cecal microbial structure at the OTU level using the “ape” package of R (version 4.0.4). An analysis of similarities (ANOSIM) with Bray–Curtis distance matrices was performed with the “vegan” package of R (version 4.0.4) to provide a metric of the degree of separation in the cecal microbial structure of voles among the three groups using the permutation test. The results of PCoA and ANOSIM were plotted using the “ggplot2” package in R (version 4.0.4). Significance was set at *P* < 0.05. The ANOVA and non-parametric Kruskal–Wallis tests were performed using IBM SPSS Statistics 22 for Windows (IBM Corp., Armonk, NY, United States).

## Results

**FIGURE 1:**
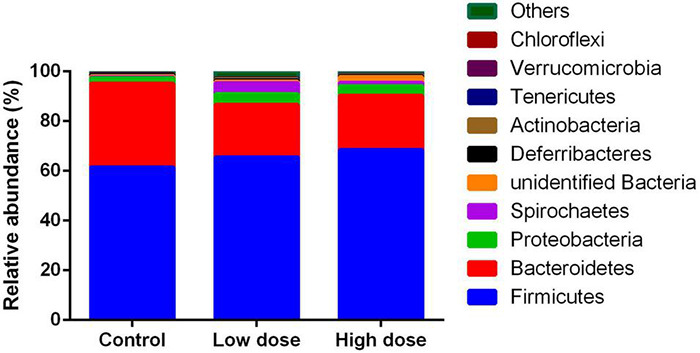
Relative abundance of operational taxonomic units among control group (0 mg/kg 6-MBOA), low 6-MBOA dose group (1 mg/kg 6-MBOA) and high 6-MBOA dose group (2 mg/kg 6-MBOA) at the phylum level in the cecal microbiota of adult male Brandt’s vole. Others mean the phyla with relative abundance less than 0.01%.

A total of 1,211,102 effective sequences were used in abundance and diversity analyses, as well as KEGG pathway comparisons. A total of 1,994 OTUs were identified, with 776-1,236 OTUs in each sample. The numbers of total tags, taxon tags, and OTUs were not significantly different among the three groups (Table 1). The average Goods coverage was as high as 99.63% among the three groups, showing that the majority of bacteria present in the samples were identified in our study (Table 1). The dominant phyla identified were Firmicutes (68.76%), followed by Bacteroidetes (22.69%), Proteobacteria (2.76%), and Spirochaetes (2.15%) (Figure 1).

**TABLE 1:** Number of total tags, taxon tags, operational taxonomic units (OTUs), and Goods coverage in 16S rRNA libraries of control group (0 mg/kg 6-MBOA), low 6-MBOA dose group (1 mg/kg 6-MBOA), and high 6-MBOA dose group (2 mg/kg 6-MBOA) in adult male Brandt’s vole (mean ± SE) (*n* = 6).

Groups	Total tags	Taxon tags	OTU numbers	Goods overage (%)
Control	80,091 ± 31	67,064 ± 818	933 ± 50	99.70 ± 0.00
Low dose	80,082 ± 17	66,915 ± 718	1,069 ± 42	99.65 ± 0.02
High dose	80,120 ± 28	67,872 ± 862	954 ± 48	99.63 ± 0.02

**FIGURE 2:**
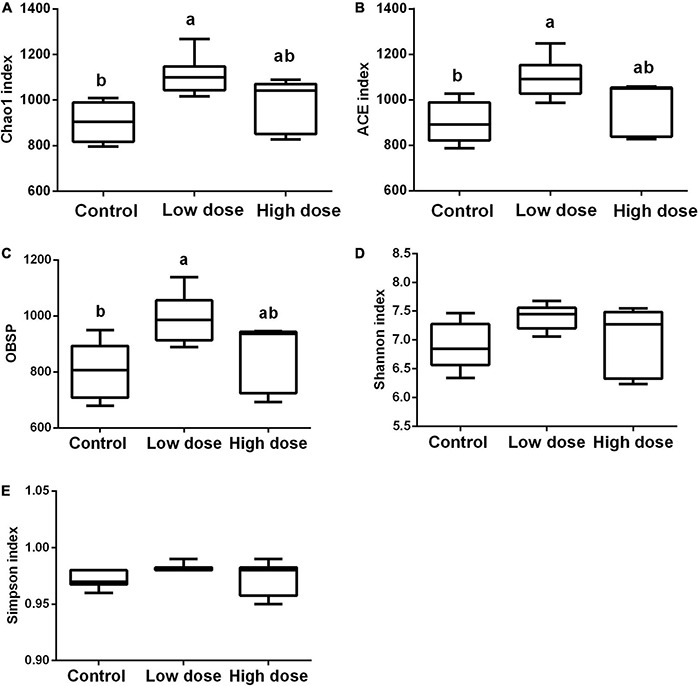
Differences in the cecal microbiome composition among control group (0 mg/kg 6-MBOA), low 6-MBOA dose group (1 mg/kg 6-MBOA) and high 6-MBOA dose group (2 mg/kg 6-MBOA) in adult male Brandt’s vole. **(A)** chao1; **(B)** abundance-based coverage estimator (ACE); **(C)** Observed species (OBSP); **(D)** Shannon index; **(E)** Simpson index. Error bars indicate standard errors. Same letters connect bars with no significant differences at *P* < 0.05 (*n* = 6).

Statistical analysis using the Kruskal-Wallis test showed that after 15 days of treatment, 6-MBOA had significant effects on Chao1 (*P* = 0.007), ACE indices (*P* = 0.016), and observed species (*P* = 0.035), with each parameter being higher in the low 6-MBOA dose group than in the control group (*P* = 0.002, 0.004, and 0.011, respectively) (Figures 2A–C). The 6-MBOA had no significant effects on the Shannon (*P* = 0.113) (Figure 2D) or Simpson indices (*P* = 0.412) (Figure 2E).

**FIGURE 3:**
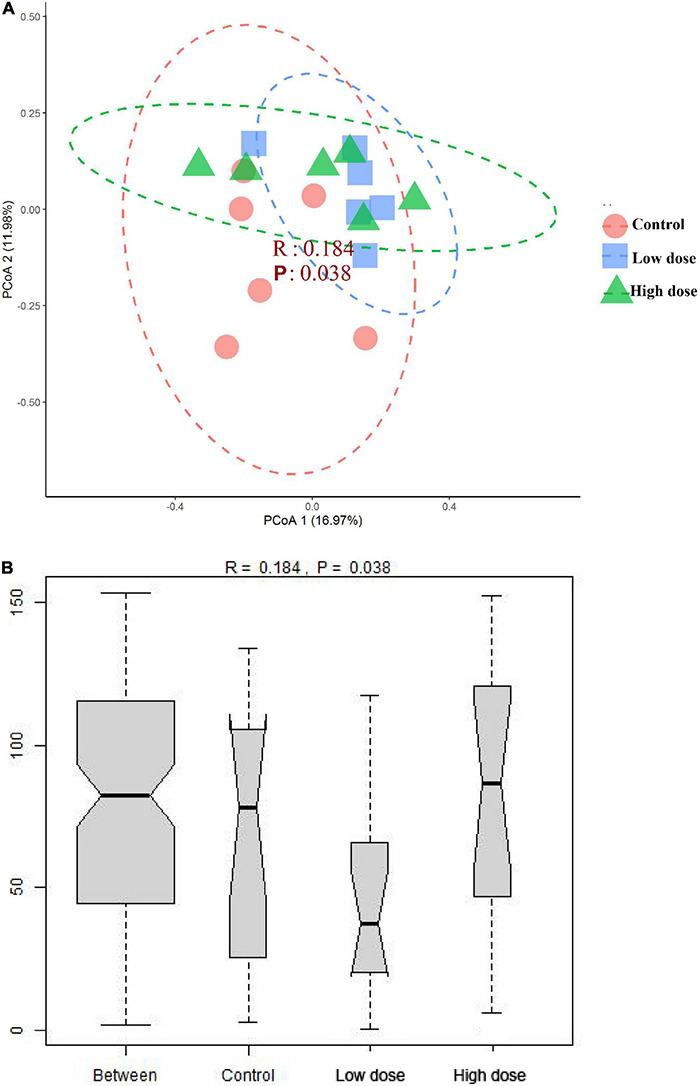
Structural separation of that cecal microbiota among control group (0 mg/kg 6-MBOA), low 6-MBOA dose group (1 mg/kg 6-MBOA) and high 6-MBOA dose group (2 mg/kg 6-MBOA) in adult male Brandt’s vole (*n* = 6). **(A)** Principal coordinates analysis (PCoA) plot; **(B)** ANOSIM analysis plot.

The PERMANOVA results showed that 6-MBOA administration for 15 days significantly altered the beta diversity of the cecal microbial community (*F* = 1.3778, *P* = 0.035), with the 6-MBOA dose group being significantly different from the control group (*P* = 0.027). Additionally, the profile of dissimilarities in the structure of the cecal microbiome after 6-MBOA administration for 15 days was depicted using PCoA of Bray–Curtis dissimilarity (Figure 3A). Principal coordinates 1 and 2 (PCo1 and PCo2) explained 16.97 and 11.98% of the variation, respectively. Although the clustering of the control, low 6-MBOA dose, and high 6-MBOA dose groups was not completely separated in the ordination space, ANOSIM revealed that the divergence in the structure of cecal microbiome between the three groups was greater than the divergence within the low 6-MBOA dose group (*R* = 0.184, *P* = 0.038) (Figure 3B).

**FIGURE 4:**
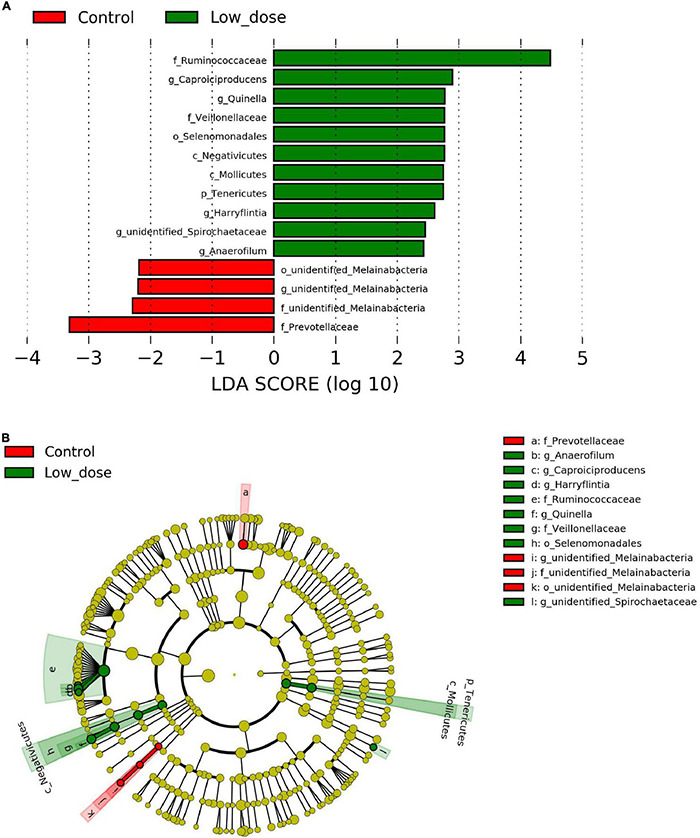
Biomarker of taxa with statistically significant differences in abundance identified in the cecal microbiota among control group (0 mg/kg 6-MBOA), low 6-MBOA dose group (1 mg/kg 6-MBOA) and high 6-MBOA dose group (2 mg/kg 6-MBOA) in adult male Brandt’s vole by linear discriminant analysis (LDA) effect size (LEfSe) analysis (*n* = 6). **(A)** Histogram showing the taxa with a LDA score significant threshold > 2. **(B)** Cladogram showing the phylogenetic position of enriched taxa among the three groups, according to the LEfSe analysis.

To identify the differential abundance and categories of the intestinal flora and to identify significantly different biomarkers among the three groups—referring to statistical tests and biological relevance—we used LEfSe (LDA Effect Size) (Score > 2). The enriched biomarkers in the low 6-MBOA dose group (compared to the control group) included the phylum Tenericutes, classes Mollicutes and Negativicutes, order Selenomonadales, families *Ruminococcaceae*, and *Veillonellaceae*, genera *Quinella*, *Caproiciproducens*, *Anaerofilum*, *Harryflintia*, and unidentified *Spirochaetaceae*. All of these belong to the phylum Firmicutes, except the Mollicutes class (phylum Tenericutes), and the unidentified genus *Spirochaetaceae* (phylum Spirochaetes) (Figures 4A,B). The enrichment of unidentified order Melainabacteria, families *Melainabacteria* and *Prevotellaceae*, and genus *Melainabacteria* was reduced in the low 6-MBOA dose group (compared to the control group).

**FIGURE 5:**
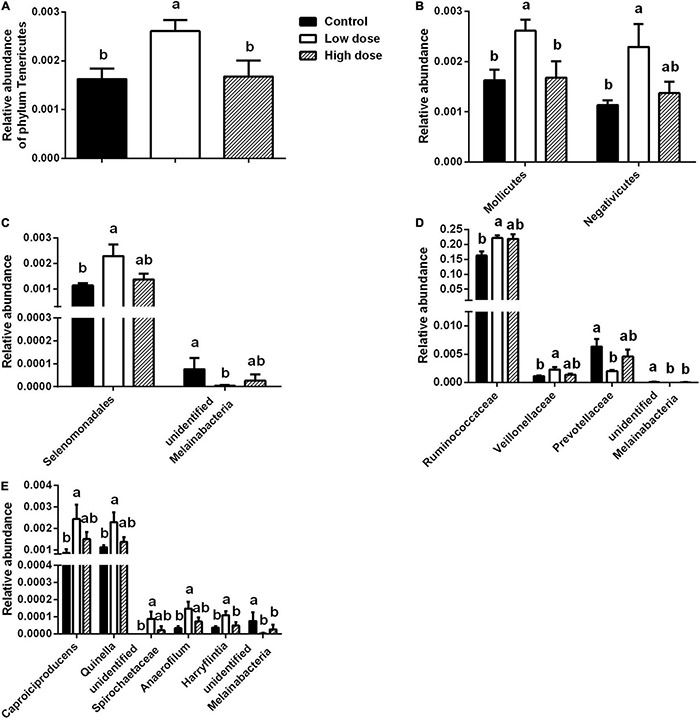
Differences in taxa abundances of cecal microbiota at different taxonomic levels among control group (0 mg/kg 6-MBOA), low 6-MBOA dose group (1 mg/kg 6-MBOA) and high 6-MBOA dose group (2 mg/kg 6-MBOA) in adult male Brandt’s vole. **(A)** Phylum level; **(B)** class level; **(C)** order level; **(D)** family level; **(E)** genus level. Error bars indicate standard errors. Same letters connect bars with no significant differences at *P* < 0.05 (*n* = 6).

Fifteen days of 6-MBOA treatment resulted in a significant change in the relative abundance of the phylum Tenericutes (*P* = 0.032), with an increase in the low 6-MBOA dose group over the control and high 6-MBOA dose groups (*P* = 0.020 and 0.030, respectively) (Figure 5A). 6-MBOA also significantly changed the relative abundance of the Mollicutes and Negativicutes classes (*P* = 0.032 and 0.043, respectively), with increases in the low 6-MBOA dose group over both the control and high 6-MBOA dose groups for the Mollicutes class (*P* = 0.020 and 0.027, respectively) (Figure 5B and Supplementary Figure 1), and just the control group for the Negativicutes class (*P* = 0.016) (Figure 5B and Supplementary Figure 1). 6-MBOA significantly altered the relative abundance of the Selenomonadales and unidentified Melainabacteria orders (*P* = 0.043 and 0.036, respectively), with an increase in the low 6-MBOA dose group over the control (*P* = 0.016) for Selenomonadales (Figure 5C and Supplementary Figure 2), and a decrease in the low 6-MBOA dose group over the control (*P* = 0.019) for unidentified Melainabacteria (Figure 5C). 6-MBOA significantly altered the relative abundance of the *Ruminococcaceae* (*P* = 0.014) and *Veillonellaceae* (*P* = 0.043) families, with an increase in the low 6-MBOA dose group over the control group (*P* = 0.008 and 0.016, respectively) (Figure 5D and Supplementary Figure 3). The relative abundance of the unidentified *Melainabacteria* family significantly altered (*P* = 0.036), with an increase in the low 6-MBOA dose group (compared to the control and high 6-MBOA dose groups) (*P* = 0.019 and 0.036, respectively) (Figure 5D). The relative abundance of the *Prevotellaceae* family was also significantly altered (*P* = 0.022) with a decrease in the low 6-MBOA dose group (compared to the control group) (*P* = 0.007) (Figure 5D and Supplementary Figure 3). Fifteen-day treatment with 6-MBOA significantly altered the relative abundance of the genera *Caproiciproducens*, *Quinella*, unidentified *Spirochaetaceae*, *Anaerofilum* and *Harryflintia* (*P* = 0.045, 0.040, 0.037, 0.044 and 0.038, respectively), with an increase in the low 6-MBOA dose group (compared to the control group) (*P* = 0.013, 0.014, 0.014, 0.013, and 0.016, respectively) (Figure 5E and Supplementary Figure 4). The relative abundance of the unidentified genus *Melainabacteria* was also significantly altered (*P* = 0.036), with a decrease in both the low and high 6-MBOA dose groups (compared to the control group) (*P* = 0.019 and 0.036, respectively) (Figure 5E).

**FIGURE 6:**
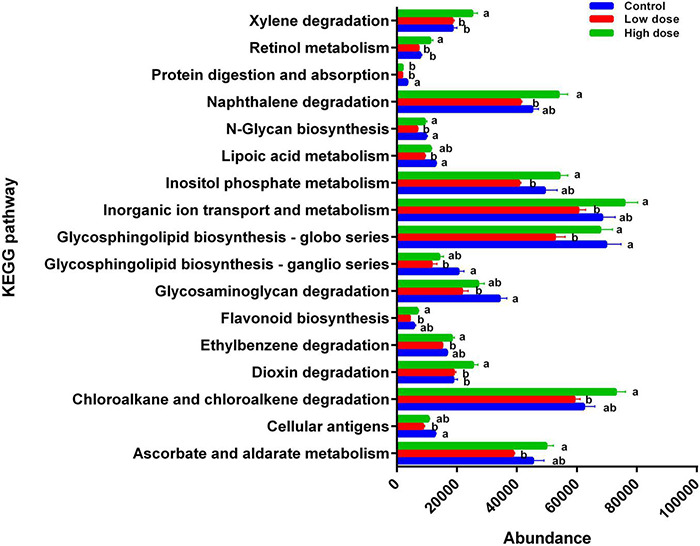
Differences in the enrichment of various KEGG pathways among control group (0 mg/kg 6-MBOA), low 6-MBOA dose group (1 mg/kg 6-MBOA) and high 6-MBOA dose group (2 mg/kg 6-MBOA) in adult male Brandt’s vole. Error bars indicate standard errors. Same letters connect bars with no significant differences at *P* < 0.05 (*n* = 6).

The functions of 17 KEGG pathways in the cecal bacterial communities changed following the administration of 6-MBOA for 15 days (Table 2 and Figure 6). Dioxin and xylene degradation (metabolism), and retinol metabolism (metabolism) were more dominant in the cecal bacterial community in the high 6-MBOA dose group than in the control and low 6-MBOA dose groups (*P* = 0.040, 0.008; *P* = 0.040, and 0.005; *P* = 0.045 and 0.004; respectively). Protein digestion and absorption (organismal systems) were more dominant in the control cecal bacterial community than in the low and high 6-MBOA dose groups (*P* = 0.017 and 0.045, respectively). Cellular antigens (environmental information processing) (*P* = 0.002), glycosaminoglycan degradation (metabolism) (*P* = 0.003), glycosphingolipid biosynthesis-ganglio series (metabolism) (*P* = 0.007), glycosphingolipid biosynthesis-globo series (metabolism) (*P* = 0.027), N-glycan biosynthesis (metabolism) (*P* = 0.005), and lipoic acid metabolism (metabolism) (*P* = 0.003) were more dominant in the control cecal bacterial community than in the low 6-MBOA dose group. Ascorbate and aldarate metabolism (metabolism) (*P* = 0.013), chloroalkane and chloroalkene degradation (metabolism) (*P* = 0.008), ethylbenzene degradation (metabolism) (*P* = 0.004), flavonoid biosynthesis (metabolism) (*P* = 0.005), inorganic ion transport and metabolism (unclassified) (*P* = 0.011), inositol phosphate metabolism (metabolism) (*P* = 0.009), N-glycan biosynthesis (metabolism) (*P* = 0.027), glycosphingolipid biosynthesis-globo series (metabolism) (*P* = 0.031), and naphthalene degradation (metabolism) (*P* = 0.002) were more dominant in the cecal bacterial community in the high 6-MBOA dose group than in the low 6-MBOA dose group.

**TABLE 2:** KEGG pathways with significantly varied enrichment in the cecal microbiota of adult male Brandt’s vole in response to the administration of 6-MBOA (*n* = 6).

KEGG pathways	*P*-value
Ascorbate and aldarate metabolism (Metabolism)	0.039
Cellular antigens (Environmental information processing)	0.008
Chloroalkane and chloroalkene degradation (Metabolism)	0.029
Dioxin degradation (Metabolism)	0.021
Ethylbenzene degradation (Metabolism)	0.015
Flavonoid biosynthesis (Metabolism)	0.018
Glycosaminoglycan degradation (Metabolism)	0.012
Glycosphingolipid biosynthesis—ganglio series (Metabolism)	0.022
Glycosphingolipid biosynthesis—globo series (Metabolism)	0.041
Inorganic ion transport and metabolism (Unclassified)	0.038
Inositol phosphate metabolism (Metabolism)	0.029
Lipoic acid metabolism (Metabolism)	0.012
N-Glycan biosynthesis (Metabolism)	0.012
Naphthalene degradation (Metabolism)	0.007
Protein digestion and absorption (Organismal Systems)	0.038
Retinol metabolism (Metabolism)	0.013
Xylene degradation (Metabolism)	0.014

**FIGURE 7:**
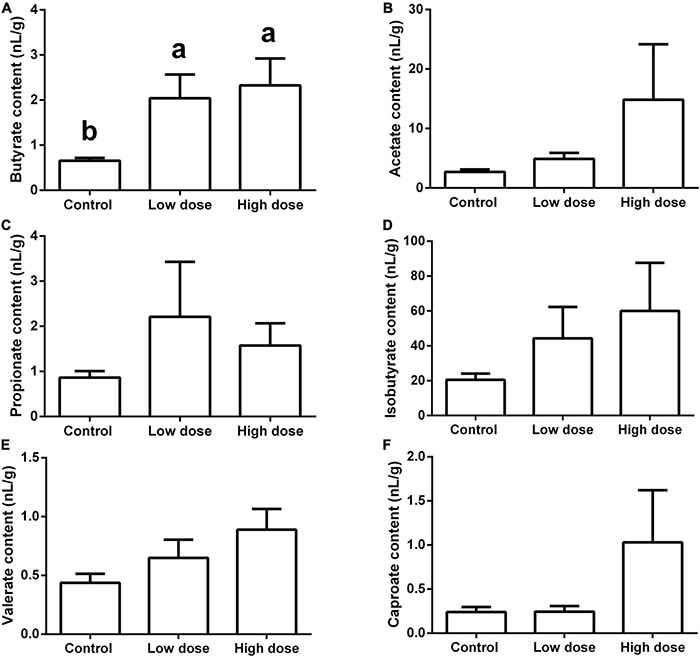
Short-chain fatty acids (SCFAs) content in the cecum of adult male Brandt’s vole intragastrically administered a 6-MBOA dose of 0 mg/kg (control group), 1 mg/kg (low dose group), or 2 mg/kg (high dose group). **(A)** Butyrate; **(B)** acetate; **(C)** propionate; **(D)** isobutyrate; **(E)** valerate; **(F)** caproate. Error bars indicate standard errors. Same letters connect bars with no significant differences at *P* < 0.05 (*n* = 8).

**FIGURE 8:**
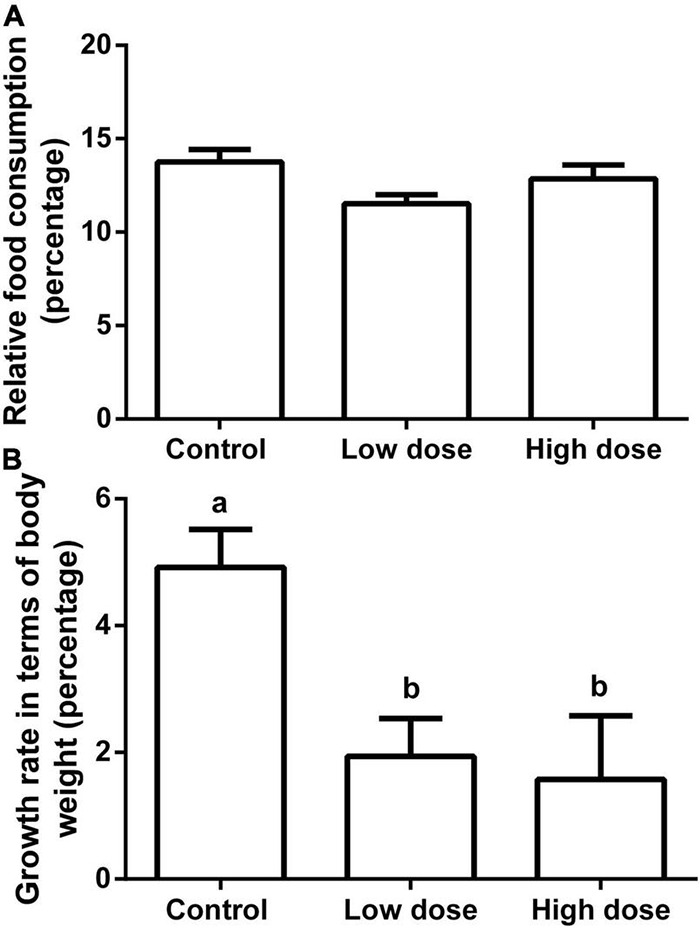
Relative food consumption and growth rate in terms of body weight of adult male Brandt’s vole intragastrically administered a 6-MBOA dose of 0 mg/kg (control group), 1 mg/kg (low dose group), or 2 mg/kg (high dose group). **(A)** Relative food consumption. **(B)** Growth rate in terms of body weight. Error bars indicate standard errors. Same letters connect bars with no significant differences at *P* < 0.05 (*n* = 8).

Fifteen days of 6-MBOA treatment significantly increased the butyrate content (*P* = 0.008) in the low and high 6-MBOA dose groups (compared to control) (*P* = 0.016, 0.027, respectively) (Figure 7A), while the contents of other five SCFAs were not significantly affected by 6-MBOA (Figures 7B–F) (*P* > 0.05). ANOVA showed that 6-MBOA administration for 15 days did not significantly affect the relative food consumption [*F*_(2, 21)_ = 3.104, *P* = 0.066] (Figure 8A), while it significantly affected the growth rate in terms of body weight in male Brandt’s voles [*F*_(2, 21)_ = 5.838, *P* = 0.010] (Figure 8B). The growth rate in the control group was significantly higher than that in the low and high 6-MBOA dose groups (*P* = 0.029, *P* = 0.014) (Figure 8B).

## Discussion

In this study, the dominant phyla were Firmicutes and Bacteroidetes. Consistently, these were also the dominant phyla in the fresh feces of wild Brandt’s voles (Li et al., 2020; Xu and Zhang, 2021). However, the ratio of Firmicutes to Bacteroidetes decreased sharply from 9.7 in the previous study (Li et al., 2020) to approximately 3.0, in the present study. The abundance of Bacteroidetes increased in the guts of captive primates when the dietary fiber content decreased (Clayton et al., 2016). In the study by Li et al. (2020), wild voles feeding on fresh grassland plants yielded a plant fiber content in the diet of approximately 30%. In our study, as the voles were fed rodent chow, which only contained approximately 5% crude fiber, the fiber content in the diet was significantly less than that in the food of wild voles. Therefore, we infer that the large decrease in the fiber content in the diet contributed to the sharp increase in the abundance of Bacteroidetes, and thus the large decrease in the Firmicutes to Bacteroidetes ratio in the gut of voles in our study in comparison to that in the wild voles in the study by Li et al. (2020).

Compared to the control group, the low 6-MBOA dose group showed an increase in observed species, Chao1, and ACE indices of cecal microbiota, as well as an enrichment in biomarker OTUs; furthermore, a separation of the cecal microbiota structure in the low 6-MBOA dose group from the control group were also observed. This suggests that 6-MBOA could enhance the abundance of microbial species in the cecum of Brandt’s vole, thus improving enrichment and altering the structure of the cecal microbiota. However, the differences in these parameters between the high 6-MBOA dose group and the control group were not significant, indicating that the effect of 6-MBOA on the cecal microbiota was dose-dependent.

In the present study, glycan biosynthesis and metabolism functions were concurrently inhibited in the low 6-MBOA dose group, including functions, such as glycosaminoglycan degradation, glycosphingolipid biosynthesis-ganglio series, glycosphingolipid biosynthesis-globo series, and N-glycan biosynthesis. Members of the *Prevotellaceae* family can activate intestinal gluconeogenesis to ameliorate glucose homeostasis (De Vadder et al., 2016). *Veillonellaceae* bacteria have been found to be associated with higher serum insulin concentrations (Cheng et al., 2018). In this study, the abundance of *Prevotellaceae* in the low 6-MBOA dose group was lower than that in the control group, whereas that of *Veillonellaceae* was higher in the low 6-MBOA dose group. 6-MBOA was reported to improve glucose tolerance in diabetic rats (Hameed et al., 2019). Thus, we infer that 6-MBOA administration can vary the enrichment of pathways pertaining to glucose metabolism by reducing the abundance of *Prevotellaceae* and increasing the abundance of *Veillonellaceae* to help regulate glucose homeostasis in voles. Enrichment of bacteria belonging to the phylum Tenericutes was found in healthy individuals (compared to that in patients with metabolic syndromes, such as those with obesity) (Lim et al., 2017). Lower abundance of *Anaerofilum* has been found in obese patients (Del Chierico et al., 2018; Koo et al., 2019). In this study, the abundance of Tenericutes and *Anaerofilum* in the low 6-MBOA dose group was higher than that in the control group. Thus, enhancement of Tenericutes and *Anaerofilum* abundance in the cecum induced by 6-MBOA may contribute to body mass control in voles.

Negativicutes bacteria can produce propionate (Hino and Kuroda, 1993). Selenomonadales bacteria belonging to the class Negativicutes have been reported to ferment carbohydrates into acetate (Vargas et al., 2017). *Veillonellaceae* bacteria belonging to the order Selenomonadales are also thought to be related to fatty acid metabolism (Zeng et al., 2019). *Quinella* bacteria belonging to the *Veillonellaceae* family can produce propionate (Kittelmann et al., 2014). Mollicutes bacteria belonging to the phylum Tenericutes also help produce acetate (Sapountzis et al., 2018). Propionate produced by microbial fermentation in the large intestine may contribute to human health maintenance (Reichardt et al., 2014). Acetate aids host substrate metabolism by enabling the secretion of gut hormones and is also involved in the regulation of glucose homeostasis and appetite (Hernández et al., 2019). In the present study, the abundance of classes Mollicutes and Negativicutes, order Selenomonadales, family *Veillonellaceae*, and genus *Quinella* was concurrently higher in the low-dose group than in the control group. Meanwhile, increased acetate and propionate content was detected in the low 6-MBOA dose group (compared to the control group), although the differences were not significant. Thus, we inferred that 6-MBOA can enhance the abundance of these bacteria in the cecum to help regulate the production of acetate and propionate and thus benefit overall health. As *Caproiciproducens* can produce butyrate (Bengelsdorf et al., 2019; Flaiz et al., 2020), its higher abundance may partially contribute to the higher butyrate content in the cecum of the low 6-MBOA dose group. Besides, butyrate-producing *Butyrivibrio* (Kim et al., 2020) and *Acetatifactor* (Pfeiffer et al., 2012) enriched in low and high 6-MBOA dose group, respectively (not significantly; Supplementary Figure 4) may also help enhance the product of butyrate in the cecum. Butyrate is beneficial to humans and animals because of its anti-inflammatory properties (Sossai, 2012). Therefore, we speculate that 6-MBOA improves the health of voles by enhancing the butyrate levels in the cecal microbiota. *Caproiciproducens, Anaerofilum*, and *Harryflintia* belong to the family *Ruminococcaceae*, and therefore the increased abundance of these three genera in the low 6-MBOA dose group contributed to the higher abundance of the *Ruminococcaceae* family in the low 6-MBOA dose group (compared to the control group).

Microbiotas in the large intestine that ferment proteins are important for protein metabolism (Xie et al., 2020). In the present study, both protein digestion and absorption were down-regulated in low and high 6-MBOA dose groups, which corresponds to inhibitory effect of 6-MBOA on protein digestion in *Ostrinia nubilalis* (Houseman et al., 1992). The abundance of the phylum Tenericutes in the ileal of pigs decreased as the dietary protein content reduced (Qiu et al., 2018). The abundance of the family *Prevotellaceae* in the rumen of *Bos indicus* decreased with the protein supplement (Latham et al., 2018). Consistently, the abundance of Tenericutes increased while that of the family *Prevotellaceae* reduced, which hints that the content of undigested protein left in the cecum by the 6-MBOA administration was high. In a parallel study, we observed that 6-MBOA significantly inhibited the *in vitro* activity of the intestinal trypsin in male Brandt’s voles and increased the nitrogen content of male Brandt’s voles feces (unpublished). Altogether, 6-MBOA could inhibit the protein digestion in Brandt’s voles. Moreover, in the 6-MBOA groups, the growth rate was reduced, with an observable food consumption decrease. Acetate, butyrate and propionate are known to stimulate the release of peptide YY inhibiting appetite (Karaki et al., 2006; Charrier et al., 2013; Hernández et al., 2019). The concentration of serum ghrelin was also reduced by 6-MBOA administration in Brandt’s voles in the parallel study (unpublished). Thus, we infer that restricted protein digestion, reduced serum ghrelin, and the enhanced SCFAs in cecum by 6-MBOA may together contribute to the reduction in food intake. Altogether these results indicate that 6-MBOA administration may lead to a reduced growth rate. Altered abundance of cecum microbiota related to glucose homeostasis regulation and body mass in response to 6-MBOA might also contribute to the reduced growth rate. Consistently, 6-MBOA-containing cereal grain products were used for obesity treatment in human (Fomsgaard et al., 2011). The restricted the protein digestion and absorption of the cecal microbiota, the decreased trend of relative food consumption, and the reduced growth rate in Brandt’s vole suggest that 6-MBOA can potentially act as a digestion-inhibiting PSM in the interaction between mammalian herbivores and plants.

Gut microbes can help the human body transform industrial chemicals and pollutants, alter their toxicities and half-lives, and are linked to health benefits (Koppel et al., 2017). Nitroreductases in the cecal contents of Sprague–Dawley rats produced by cecum microbiota can transform the xenobiotic nitrazepam (Takeno and Sakai, 1991). Woodrats (*Neotoma lepida*) consuming diets containing creosote resin (a PSM with aromatic rings) harbored microbes with a higher abundance of genes associated with the metabolism of aromatic compounds (Kohl et al., 2014). Notably, 6-MBOA also has one aromatic ring. Xylene, dioxin, naphthalene, and ethylbenzene are aromatic xenobiotics for animals. Therefore, the upregulated degradation of xylene and dioxin in the high 6-MBOA dose group (compared to that in the control group), and the enhanced degradation of dioxin, xylene, naphthalene, and ethylbenzene in the high 6-MBOA dose group (compared to that in the low 6-MBOA dose group), indicate that 6-MBOA induced a change in the metabolism of aromatic xenobiotics in the cecal bacteria and that the intensity of the change was dose-dependent. It has been reported that 6-MBOA can be transformed by fungi such as *Fusarium moniliiforme* and *Gaumannomyces graminis* (Fomsgaard et al., 2004) to N-(2-hydroxy-4-methoxyphenyl) malonamic acid (HMPMA), and caterpillars (*Spodoptera littoralis* and *S. frugiperda*) to MBOA-*N*-Glc in the gut (Maag et al., 2014). Therefore, intense metabolism of aromatic xenobiotics by cecum microbiota in the high 6-MBOA dose group might result in more metabolized 6-MBOA. This effect could partially explain why a high dose of 6-MBOA did not significantly influence the cecal microbiota community and indicate that the cecum microbiota can metabolize 6-MBOA. This also implies that a high dose of 6-MBOA might be harmful to voles due to the reinforced protective mechanism of xenobiotics in the cecal microbiota. We infer that the abundance of bacteria enhanced by 6-MBOA in the present study should be 6-MBOA tolerant or involved in the 6-MBOA metabolism. However, the exact cecal bacteria and mechanism by which 6-MBOA is metabolized in Brandt’s voles need more in-depth research. Reduced abundances in unidentified Melainabacteria, family of the unidentified *Melainabacteria* and *Prevotellaceae*, and the unidentified genus *Melainabacteria* suggest that those bacteria are susceptible to the administration of 6-MBOA, which verifies the antimicrobial properties of 6-MBOA (Wang et al., 2001; Wang and Ng, 2002; Martyniuk et al., 2006). Chloroalkane and chloroalkene degradation, a pathway also involved in xenobiotic biodegradation and metabolism (Liu F. et al., 2021), was consistently observed to be dose-dependently improved by 6-MBOA administration in cecal microbiota of Brandt’s voles.

High doses of 6-MBOA, which exerts negative effects on the growth, food consumption, and protein digestion and absorption of cecal microbiota, and stimulates the degradation of xenobiotics in cecal microbiota without improving enrichment or altering the structure, hint that daily ingestion of more than 100 μg of 6-MBOA may diminish its benefit and exert potential harmful effect on Brandt’s voles. *Desulfovibrio* is known as sulfate-reducing bacteria, which is responsible for infections and diarrheas in mammals (Velasco-Galilea et al., 2020). *Helicobacter* is associated with severe gastric disease (Ferreira et al., 2018). Decrease in *Roseburia* abundance may damage various metabolic pathways and incur irritable bowel syndrome (Tamanai-Shacoori et al., 2017). The abundance of *Desulfovibrio* and *Helicobacter* enhanced and *Roseburia* reduced in high 6-MBOA dose group (not significantly, Supplementary Figure 4) furthermore indicates that high dose of 6-MBOA may impair the health of Brandt’s voles. Consistently, further intraperitoneal injection of 6-MBOA conversely weakened its stimulating effect on the reproduction of male Brandt’s vole under short photoperiods (Dai et al., 2016). Thus, we propose that 6-MBOA could protect *L. chinensis* from Brandt’s voles if the daily 6-MBOA intake dramatically exceeds 100 μg. Further ingestion of 6-MBOA by *L. chinensis* may impair voles in grasslands. These may indicate that 6-MBOA could act as a defensive secondary metabolite against mammalian herbivores in *L. chinensis* seedlings (Dai et al., 2014). In contrast, our results demonstrate that cecal bacteria can assist Brandt’s voles in eliminating ingested xenobiotics, which confirms the detoxifying strategy of the gut microbiota during the adaptation of herbivores to PSMs in the coevolution between plants and herbivores (Dearing et al., 2005).

In summary, our study demonstrated that 6-MBOA altered the enrichment of the microbial community and the abundance of bacteria in the cecum of Brandt’s voles, thereby inducing changes in the community structure of the cecal microbiota, SCFA content, and pathway enrichment. 6-MBOA mainly affected functions related to glucose metabolism, protein digestion and absorption, and body weight control of cecal microbiota in male Brandt’s voles. Metabolism of xenobiotics dose-dependently stimulated by 6-MBOA hints that cecal microbiota can aid the elimination of ingested xenobiotics in male Brandt’s voles. The digestive functions of the cecal microbiota and body growth rate restriction due to 6-MBOA administration suggest that in addition to its role as a reproduction stimulator in mammalian herbivores, 6-MBOA can potentially act as a digestion-inhibiting (PSMs) in the interaction between mammalian herbivores and plants.

## Data Availability Statement

The original contributions presented in the study are included in the article/Supplementary Material, further inquiries can be directed to the corresponding author/s.

## Ethics Statement

The animal study was reviewed and approved by the Animal Care and Use Committee of the Faculty of Veterinary Medicine of Yangzhou University.

## Author Contributions

WW supplied the funding and designed the experiment. SY supplied the funding and revised the manuscript. XD performed the statistical analyses and wrote the manuscript. LC, ML, YL, SJ, and TX performed the experiment. AW provided technological support for this experiment. All authors contributed to the article and approved the submitted version.

## Conflict of Interest

The authors declare that the research was conducted in the absence of any commercial or financial relationships that could be construed as a potential conflict of interest.

## Publisher’s Note

All claims expressed in this article are solely those of the authors and do not necessarily represent those of their affiliated organizations, or those of the publisher, the editors and the reviewers. Any product that may be evaluated in this article, or claim that may be made by its manufacturer, is not guaranteed or endorsed by the publisher.

## Footnotes

1
https://www.arb-silva.de/

2
https://submit.ncbi.nlm.nih.gov/subs/bioproject/

3
http://huttenhower.sph.harvard.edu/galaxy/

## Supplementary Material

Supplementary Figure 1 Relative abundance of operational taxonomic units among control group (0 mg/kg 6-MBOA), low 6-MBOA dose group (1 mg/kg 6-MBOA) and high 6-MBOA dose group (2 mg/kg 6-MBOA) at the class level in the cecal microbiota of adult male Brandt’s vole. Others mean the classes with relative abundance less than 0.1%.

Supplementary Figure 2 Relative abundance of operational taxonomic units among control group (0 mg/kg 6-MBOA), low 6-MBOA dose group (1 mg/kg 6-MBOA) and high 6-MBOA dose group (2 mg/kg 6-MBOA) at the order level in the cecal microbiota of adult male Brandt’s vole. Others mean the orders with relative abundance less than 0.1%.

Supplementary Figure 3 Relative abundance of operational taxonomic units among control group (0 mg/kg 6-MBOA), low 6-MBOA dose group (1 mg/kg 6-MBOA) and high 6-MBOA dose group (2 mg/kg 6-MBOA) at the family level in the cecal microbiota of adult male Brandt’s vole. Others mean the families with relative abundance less than 0.1%.

Supplementary Figure 4 Relative abundance of operational taxonomic units among control group (0 mg/kg 6-MBOA), low 6-MBOA dose group (1 mg/kg 6-MBOA) and high 6-MBOA dose group (2 mg/kg 6-MBOA) at the genus level in the cecal microbiota of adult male Brandt’s vole. Others mean the genera with relative abundance less than 0.1%.

## References

[B1] Acharya J. Kaspar T. C. Robertson A. E. (2021). Effect of 6-Methoxy-2-Benzoxazolinone (MBOA) on *Pythium* species and corn seedling growth and disease. *Plant Dis.* 105 752–757. 10.1094/PDIS-04-20-0824-SC 33048595

[B2] Alibhai S. K. (1986). Reproductive response of *Gerbillus harwoodii* to 6-MBOA in Kora National Reserve, Kenya. *J. Trop. Ecol.* 2 377–379. 10.1017/S0266467400001012

[B3] Anderson K. D. Nachman R. J. Turek F. W. (1988). Effects of melatonin and 6-methoxybenzoxazolinone on photoperiodic control of testis size in adult male golden hamsters. *J. Pineal Res.* 5 351–365. 10.1111/j.1600-079x 3210136

[B4] Argandoña V. H. Niemeyer H. M. Corcuera L. J. (1981). Effect of content and distribution of hydroxamic acids on infestation in the aphid *Schizaphis graminum*. *Phytochemistry* 20 673–676. 10.1016/0031-9422(81)85154-0

[B5] Bengelsdorf F. R. Poehlein A. Daniel R. Dürre P. (2019). Genome sequence of the caproic acid-producing bacterium *Caproiciproducens galactitolivorans* BS-1^T^ (JCM 30532). *Microbiol. Resour. Announc.* 8:e346-19. 10.1128/MRA.00346-19 31371534 PMC6675982

[B6] Berger P. J. Negus N. C. Sanders E. H. Gardner P. D. (1981). Chemical triggering of reproduction in *Microtus montanus*. *Science* 214 69–70. 10.1126/science.7025210 7025210

[B7] Bo T. B. Zhang X. Y. Wen J. Deng K. Qin X. W. Wang D. H. (2019). The microbiota-gut-brain interaction in regulating host metabolic adaptation to cold in male Brandt’s voles (*Lasiopodomys brandtii*). *ISME J.* 13 3037–3053. 10.1038/s41396-019-0492-y 31455805 PMC6863827

[B8] Campos F. Atkinson J. Arnason J. T. Philogéne B. J. Morand P. Werstiuk N. H. (1988). Toxicity and toxicokinetics of 6-methoxybenzoxazolinone (MBOA) in the european corn borer, *Ostrinia nubilalis* (Hübner). *J. Chem. Ecol.* 14 989–1002. 10.1007/BF01018788 24276146

[B9] Charrier J. A. Martin R. J. McCutcheon K. L. Raggio A. M. Goldsmith F. Goita M. (2013). High fat diet partially attenuates fermentation responses in rats fed resistant starch from high-amylose maize. *Obesity* 21 2350–2355. 10.1002/oby.20362 23512798 PMC5225625

[B10] Chen X. He D. Zhou L. Cao Y. Li Z. (2020). Influence of hydropower stations on the water microbiota in the downstream of Jinsha River, China. *PeerJ* 8:e9500. 10.7717/peerj.9500 32742790 PMC7369022

[B11] Cheng J. Xue F. Zhang M. Cheng C. Qiao L. Ma J. (2018). TRIM31 deficiency is associated with impaired glucose metabolism and disrupted gut microbiota in mice. *Front. Physiol.* 9:24. 10.3389/fphys.2018.00024 29497383 PMC5818424

[B12] Clayton J. B. Vangay P. Huang H. Ward T. Hillmann B. M. Al-Ghalith G. A. (2016). Captivity humanizes the primate microbiome. *Proc. Natl. Acad. Sci. U.S.A.* 113 10376–10381. 10.1073/pnas.1521835113 27573830 PMC5027417

[B13] Dai X. Jiang L. Y. Han M. Ye M. H. Wang A. Q. Wei W. H. (2016). Reproductive responses of male Brandt’s voles (*Lasiopodomys brandtii*) to 6 -methoxybenzoxazolinone (6-MBOA) under short photoperiod. *Naturwissenschaften* 103:29. 10.1007/s00114-016-1347-2 26940061

[B14] Dai X. Zhang Y. Q. Jiang L. Y. Yuan F. Wang A. Q. Wei W. H. (2014). Evaluation of the variations in secondary metabolite concentrations of *Leymus chinensis* seedlings. *Isr. J. Ecol. Evol.* 60 75–84. 10.1080/15659801.2014.986878

[B15] De Filippo C. Cavalieri D. Di Paola M. Ramazzotti M. Poullet J. B. Massart S. (2010). Impact of diet in shaping gut microbiota revealed by a comparative study in children from Europe and rural Africa. *Proc. Natl. Acad. Sci. U.S.A.* 107 14691–14696. 10.1073/pnas.1005963107 20679230 PMC2930426

[B16] De Vadder F. Kovatcheva-Datchary P. Zitoun C. Duchampt A. Bäckhed F. Mithieux G. (2016). Microbiota-produced succinate improves glucose homeostasis via intestinal gluconeogenesis. *Cell Metab.* 24 151–157. 10.1016/j.cmet.2016.06.013 27411015

[B17] Dearing M. D. Foley W. J. McLean S. (2005). The influence of plant secondary metabolites on the nutritional ecology of herbivorous terrestrial vertebrates. *Annu. Rev. Ecol. Evol. Syst.* 36 169–189. 10.1146/annurev.ecolsys.36.102003.152617

[B18] Del Chierico F. Abbatini F. Russo A. Quagliariello A. Reddel S. Capoccia D. (2018). Gut microbiota markers in obese adolescent and adult patients: age-dependent differential patterns. *Front. Microbiol.* 9:1210. 10.3389/fmicb.2018.01210 29922272 PMC5996250

[B19] Dowd P. F. Vega F. E. (1996). Enzymatic oxidation products of allelochemicals as a basis for resistance against insects: effects on the corn leafhopper *Dalbulus maidis*. *Nat. Toxins* 4 85–91. 10.1002/19960402nt5 8726328

[B20] Edgar R. C. (2013). UPARSE: highly accurate OTU sequences from microbial amplicon reads. *Nat. Methods* 10 996–998. 10.1038/nmeth.2604 23955772

[B21] Edgar R. C. Haas B. J. Clemente J. C. Quince C. Knight R. (2011). UCHIME improves sensitivity and speed of chimera detection. *Bioinformatics* 27 2194–2200. 10.1093/bioinformatics/btr381 21700674 PMC3150044

[B22] Ferreira R. M. Pereira-Marques J. Pinto-Ribeiro I. Costa J. L. Carneiro F. Machado J. C. (2018). Gastric microbial community profiling reveals a dysbiotic cancer-associated microbiota. *Gut* 67 226–236. 10.1136/gutjnl-2017-314205 29102920 PMC5868293

[B23] Flaiz M. Baur T. Brahner S. Poehlein A. Daniel R. Bengelsdorf F. R. (2020). *Caproicibacter fermentans* gen. nov., sp. nov., a new caproate-producing bacterium and emended description of the genus *Caproiciproducens*. *Int. J. Syst. Evol. Microbiol.* 70 4269–4279. 10.1099/ijsem.0.004283 32584751

[B24] Fomsgaard I. S. Mortensen A. G. Carlsen S. C. (2004). Microbial transformation products of benzoxazolinone and benzoxazinone allelochemicals–a review. *Chemosphere* 54 1025–1038. 10.1016/j.chemosphere.2003.09.044 14664831

[B25] Fomsgaard I. S. Mortensen A. G. Holm P. B. Gregersen P. L. (2011). *Use of Benzoxazinoids-Containing Cereal Grain Products for Health-Improving Purposes.* U.S. Patent No 0020480. Washington, DC: U.S. Patent and Trademark Office.

[B26] Freeland W. J. Janzen D. H. (1974). Strategies in herbivory by mammals: the role of plant secondary compounds. *Am. Nat.* 269–289. 10.1086/282907

[B27] Haas B. J. Gevers D. Earl A. M. Feldgarden M. Ward D. V. Giannoukos G. (2011). Chimeric 16S rRNA sequence formation and detection in Sanger and 454-pyrosequenced PCR amplicons. *Genome Res.* 21 494–504. 10.1101/gr.112730.110 21212162 PMC3044863

[B28] Hameed A. Hafizur R. M. Khan M. I. Jawed A. Wang H. Zhao M. (2019). Coixol amplifies glucose-stimulated insulin secretion via cAMP mediated signaling pathway. *Eur. J. Pharmacol.* 858 172514. 10.1016/j.ejphar.2019.172514 31265841

[B29] Hernández M. A. G. Canfora E. E. Jocken J. W. E. Blaak E. E. (2019). The short-chain fatty acid acetate in body weight control and insulin sensitivity. *Nutrients* 11:1943. 10.3390/nu11081943 31426593 PMC6723943

[B30] Hino T. Kuroda S. (1993). Presence of lactate dehydrogenase and lactate racemase in *Megasphaera elsdenii* grown on glucose or lactate. *Appl. Environ. Microbiol.* 59 255–259. 10.1128/aem.59.1.255-259.1993 8439152 PMC202087

[B31] Houseman J. G. Campos F. Thie N. M. R. Philogene B. J. R. Atkinson J. Morand P. (1992). Effect of the maize-derived compounds DIMBOA and MBOA on growth and digestive processes of European corn borer (Lepidoptera: Pyralidae). *J. Econ. Entomol.* 85 669–674. 10.1093/jee/85.3.669

[B32] Hu L. Robert C. A. M. Cadot S. Zhang X. Ye M. Li B. (2018). Root exudate metabolites drive plant-soil feedbacks on growth and defense by shaping the rhizosphere microbiota. *Nat. Commun.* 9:2738. 10.1038/s41467-018-05122-7 30013066 PMC6048113

[B33] Hughes C. L. Jr. (1988). Phytochemical mimicry of reproductive hormones and modulation of herbivore fertility by phytoestrogens. *Environ. Health Perspect.* 78, 171–4. 10.1289/ehp.8878171 3203635 PMC1474615

[B34] Johnson R. N. O’Meally D. Chen Z. Etherington G. J. Ho S. Y. W. Nash W. J. (2018). Adaptation and conservation insights from the koala genome. *Nat. Genet.* 50 1102–1111. 10.1038/s41588-018-0153-5 29967444 PMC6197426

[B35] Jones R. J. Megarrity R. G. (1986). Successful transfer of DHP-degrading bacteria from Hawaiian goats to Australian ruminants to overcome the toxicity of Leucaena. *Aust. Vet. J.* 63 259. 10.1111/j.1751-0813.1986.tb02990.x 3790013

[B36] Karaki S. Mitsui R. Hayashi H. Kato I. Sugiya H. Iwanaga T. (2006). Short-chain fatty acid receptor, GPR43, is expressed by enteroendocrine cells and mucosal mast cells in rat intestine. *Cell Tissue Res.* 324 353–360. 10.1007/s00441-005-0140-x 16453106

[B37] Kim S. Rigatto K. Gazzana M. B. Knorst M. M. Richards E. M. Pepine C. J. (2020). Altered gut microbiome profile in patients with pulmonary arterial hypertension. *Hypertension* 75 1063–1071. 10.1161/HYPERTENSIONAHA.119.14294 32088998 PMC7067661

[B38] Kittelmann S. Pinares-Patiño C. S. Seedorf H. Kirk M. R. Ganesh S. McEwan J. C. (2014). Two different bacterial community types are linked with the low-methane emission trait in sheep. *PLoS One* 9:e103171. 10.1371/journal.pone.0103171 25078564 PMC4117531

[B39] Klun J. A. Brindley T. A. (1966). Role of 6-methoxybenzoxazolinone in inbred resistance of host plant (maize) to first-brood larvae of European corn borer. *J. Econ. Entomol.* 59 711–718.

[B40] Koh A. De Vadder F. Kovatcheva-Datchary P. Bäckhed F. (2016). From dietary fiber to host physiology: short-chain fatty acids as key bacterial metabolites. *Cell* 165 1332–1345. 10.1016/j.cell.2016.05.041 27259147

[B41] Kohl K. D. Weiss R. B. Cox J. Dale C. Dearing M. D. (2014). Gut microbes of mammalian herbivores facilitate intake of plant toxins. *Ecol. Lett.* 17 1238–1246. 10.1111/ele.12329 25040855

[B42] Koo S. H. Chu C. W. Khoo J. J. C. Cheong M. Soon G. H. Ho E. X. P. (2019). A pilot study to examine the association between human gut microbiota and the host’s central obesity. *JGH Open* 3 480–487. 10.1002/jgh3.12184 31832548 PMC6891071

[B43] Koppel N. Maini Rekdal V. Balskus E. P. (2017). Chemical transformation of xenobiotics by the human gut microbiota. *Science* 356:eaag2770. 10.1126/science.aag2770 28642381 PMC5534341

[B44] Latham E. A. Weldon K. K. Wickersham T. A. Coverdale J. A. Pinchak W. E. (2018). Responses in the rumen microbiome of *Bos taurus* and *indicus* steers fed a low-quality rice straw diet and supplemented protein. *J. Anim. Sci.* 96 1032–1044. 10.1093/jas/sky023 29617868 PMC6093561

[B45] Li G. Li J. Kohl K. D. Yin B. Wei W. Wan X. (2019). Dietary shifts influenced by livestock grazing shape the gut microbiota composition and co-occurrence networks in a local rodent species. *J. Anim. Ecol.* 88 302–314. 10.1111/1365-2656.12920 30381827

[B46] Li G. Yin B. Li J. Wang J. Wei W. Bolnick D. I. (2020). Host-microbiota interaction helps to explain the bottom-up effects of climate change on a small rodent species. *ISME J.* 14 1795–1808. 10.1038/s41396-020-0646-y 32313262 PMC7305154

[B47] Lim M. Y. You H. J. Yoon H. S. Kwon B. Lee J. Y. Lee S. (2017). The effect of heritability and host genetics on the gut microbiota and metabolic syndrome. *Gut* 66 1031–1038. 10.1136/gutjnl-2015-311326 27053630

[B48] Liu F. Xu X. Chao L. Chen K. Shao A. Sun D. (2021). Alteration of the gut microbiome in chronic kidney disease patients and its association with serum free immunoglobulin light chains. *Front. Immunol.* 12:609700. 10.3389/fimmu.2021.609700 33868230 PMC8047322

[B49] Liu J. Huang S. Li G. Zhao J. Lu W. Zhang Z. (2020). High housing density increases stress hormone- or disease-associated fecal microbiota in male Brandt’s voles (*Lasiopodomys brandtii*). *Horm. Behav.* 126:104838. 10.1016/j.yhbeh.2020.104838 32791065

[B50] Liu J. Huang S. Zhang X. Li G. Batsuren E. Lu W. (2021). Gut microbiota reflect the crowding stress of space shortage, physical and non-physical contact in Brandt’s voles *(Lasiopodomys brandtii*). *Microbiol. Res.* 255:126928. 10.1016/j.micres.2021.126928 34883384

[B51] Liu T. H. Zhang C. Y. Din A. U. Li N. Wang Q. Yu J. Z. (2020). Bacterial association and comparison between lung and intestine in rats. *Biosci. Rep.* 40:BSR20191570. 10.1042/BSR20191570 32323724 PMC7189363

[B52] Maag D. Dalvit C. Thevenet D. Köhler A. Wouters F. C. Vassão D. G. (2014). 3-β-D-Glucopyranosyl-6-methoxy-2-benzoxazolinone (MBOA-N-Glc) is an insect detoxification product of maize 1,4-benzoxazin-3-ones. *Phytochemistry* 102 97–105. 10.1016/j.phytochem.2014.03.018 24713572

[B53] Martin L. B. Johnson E. M. Hutch C. R. Nelson R. J. (2008). 6-MBOA affects testis size, but not delayed-type hypersensitivity, in white-footed mice (*Peromyscus leucopus*). *Comp. Biochem. Physiol. A Mol. Integr. Physiol.* 149 181–187. 10.1016/j.cbpa.2007.11.006 18160321 PMC2265420

[B54] Martin M. (2011). Cutadapt removes adapter sequences from high-throughput sequencing reads. *EMBnet J.* 17 10–12. 10.14806/ej.17.1.200

[B55] Martyniuk S. Stochmal A. Macías F. A. Marín D. Oleszek W. (2006). Effects of some benzoxazinoids on *in vitro* growth of *Cephalosporium gramineum* and other fungi pathogenic to cereals and on *Cephalosporium* stripe of winter wheat. *J. Agric. Food Chem.* 54 1036–1039. 10.1021/jf050901x 16478214

[B56] Nelson R. J. Shiber J. R. (1990). Photoperiod affects reproductive responsiveness to 6-methoxy-2-benzoxazolinone in house mice. *Biol. Reprod.* 43 586–591. 10.1095/biolreprod43.4.586 2289012

[B57] Niemeyer H. M. (1988). Hydroxamic acids (4-hydroxy-1,4-benzoxazin-3-ones), defence chemicals in the gramineae. *Phytochemistry* 27 3349–3358. 10.1016/0031-9422(88)80731-3

[B58] Pfeiffer N. Desmarchelier C. Blaut M. Daniel H. Haller D. Clavel T. (2012). Acetatifactor muris gen. nov., sp. nov., a novel bacterium isolated from the intestine of an obese mouse. *Arch. Microbiol.* 194 901–907. 10.1007/s00203-012-0822-1 22659832

[B59] Qiu K. Zhang X. Jiao N. Xu D. Huang C. Wang Y. (2018). Dietary protein level affects nutrient digestibility and ileal microbiota structure in growing pigs. *Anim. Sci. J.* 89 537–546. 10.1111/asj.12946 29271556

[B60] Quast C. Pruesse E. Yilmaz P. Gerken J. Schweer T. Yarza P. (2013). The SILVA ribosomal RNA gene database project: improved data processing and web-based tools. *Nucleic Acids Res.* 41 D590–D596. 10.1093/nar/gks1219 23193283 PMC3531112

[B61] Reichardt N. Duncan S. H. Young P. Belenguer A. McWilliam Leitch C. Scott K. P. (2014). Phylogenetic distribution of three pathways for propionate production within the human gut microbiota. *ISME J.* 8 1323–1335. 10.1038/ismej.2014.14 24553467 PMC4030238

[B62] Rodríguez-De Lara R. Herrera-Corredor C. A. Fallas-López M. Rangel-Santos R. Mariscal-Aguayo V. Martínez-Hernández P. A. (2007). Influence of supplemental dietary sprouted wheat on reproduction in artificially inseminated doe rabbits. *Anim. Reprod. Sci.* 99 145–155. 10.1016/j.anireprosci.2006.04.055 16720084

[B63] Sapountzis P. Zhukova M. Shik J. Z. Schiott M. Boomsma J. J. (2018). Reconstructing the functions of endosymbiotic Mollicutes in fungus-growing ants. *eLife* 7:e39209. 10.7554/eLife.39209 30454555 PMC6245734

[B64] Shi D. Z. Hai S. Z. Lu D. Liu X. F. (1999). The structure and order in colony of Brandt’s vole. *Acta Theriol. Sin.* 19 48–55.

[B65] Sommer F. Bäckhed F. (2016). Know your neighbor: microbiota and host epithelial cells interact locally to control intestinal function and physiology. *Bioessays* 38 455–464. 10.1002/bies.201500151 26990415

[B66] Sossai P. (2012). Butyric acid: What is the future for this old substance? *Swiss Med. Wkly.* 142:w13596. 10.4414/smw.2012.13596 22674349

[B67] Sundset M. A. Barboza P. S. Green T. K. Folkow L. P. Blix A. S. Mathiesen S. D. (2010). Microbial degradation of usnic acid in the reindeer rumen. *Naturwissenschaften* 97 273–278. 10.1007/s00114-009-0639-1 20033122

[B68] Takeno S. Sakai T. (1991). Involvement of the intestinal microflora in nitrazepam-induced teratogenicity in rats and its relationship to nitroreduction. *Teratology* 44 209–214. 10.1002/tera.1420440209 1925980

[B69] Tamanai-Shacoori Z. Smida I. Bousarghin L. Loreal O. Meuric V. Fong S. B. (2017). *Roseburia* spp.: A marker of health? *Future Microbiol.* 12 157–170. 10.2217/fmb-2016-0130 28139139

[B70] Vargas J. E. Andreìs S. Snelling T. J. López-Ferreras L. Yáñez-Ruíz D. R. García-Estrada C. (2017). Effect of sunflower and marine oils on ruminal microbiota, *in vitro* fermentation and digesta fatty acid profile. *Front. Microbiol.* 8:1124. 10.3389/fmicb.2017.01124 28676798 PMC5476686

[B71] Velasco-Galilea M. Guivernau M. Piles M. Viñas M. Rafel O. Sánchez A. (2020). Breeding farm, level of feeding and presence of antibiotics in the feed influence rabbit cecal microbiota. *Anim. Microbiome* 2:40. 10.1186/s42523-020-00059-z 33499975 PMC7807820

[B72] Wan X. R. Wang M. J. Wang G. H. Liu W. Zhong W. Q. (2002a). The reproductive parameters in the marked populations of Brandt’s vole. *Acta Theriol. Sin.* 22 116–122. 10.3969/j.issn.1000-1050.2002.02.005

[B73] Wan X. R. Liu W. Wang G. H. Wang M. J. Zhong W. Q. (2002b). Relationship between the daily food consumption, cumulative food consumption and the age of *Microtus brandtii*. *Chin. J. Ecol.* 21 15–17.

[B74] Wang G. M. Zhou Q. Q. Zhong W. Q. Wang G. H. (1992). Food habits of Brandt’s vole (*Microtus brandtii*). *Acta Theriol. Sin.* 12 57–64.

[B75] Wang H. X. Liu F. Ng T. B. (2001). Examination of pineal indoles and 6-methoxy-2-benzoxazolinone for antioxidant and antimicrobial effects. *Comp. Biochem. Physiol. C Toxicol. Pharmacol.* 130 379–388. 10.1016/s1532-0456(01)00264-2 11701394

[B76] Wang H. X. Ng T. B. (2002). Demonstration of antifungal and anti-human immunodeficiency virus reverse transcriptase activities of 6-methoxy-2-benzoxazolinone and antibacterial activity of the pineal indole 5-methoxyindole-3-acetic acid. *Comp. Biochem. Physiol. C Toxicol. Pharmacol.* 132 261–268. 10.1016/s1532-0456(02)00071-6 12106902

[B77] Xie X. M. Sun R. Y. Fang J. M. (1994). The mating system and reproduction of Brandt’s voles (*Microtus brandtii*). *Acta Zool. Sin.* 40 262–265.

[B78] Xie Y. Wang C. Zhao D. Zhou G. Li C. (2020). Processing method altered mouse intestinal morphology and microbial composition by affecting digestion of meat proteins. *Front. Microbiol.* 11:511. 10.3389/fmicb.2020.00511 32322243 PMC7156556

[B79] Xu X. Zhang Z. B. (2021). Sex- and age-specific variation of gut microbiota in Brandt’s voles. *PeerJ* 9:e11434. 10.7717/peerj.11434 34164232 PMC8194415

[B80] Zeng H. Guo C. Sun D. Seddik H. E. Mao S. (2019). The ruminal microbiome and metabolome alterations associated with diet-Induced milk fat depression in dairy cows. *Metabolites* 9:154. 10.3390/metabo9070154 31340604 PMC6680951

[B81] Zhang X. Y. Sukhchuluun G. Bo T. B. Chi Q. S. Yang J. J. Chen B. (2018). Huddling remodels gut microbiota to reduce energy requirements in a small mammal species during cold exposure. *Microbiome* 6:103. 10.1186/s40168-018-0473-9 29884232 PMC5994089

