# Supplementary figures and images for: Effect of 6-Methoxybenzoxazolinone on the Cecal Microbiota of Adult Male Brandt’s Vole
Source: Front Microbiol. 2022 Mar 29;13:847073. doi: 10.3389/fmicb.2022.847073 (PMC9002351; doi:10.3389/fmicb.2022.847073)

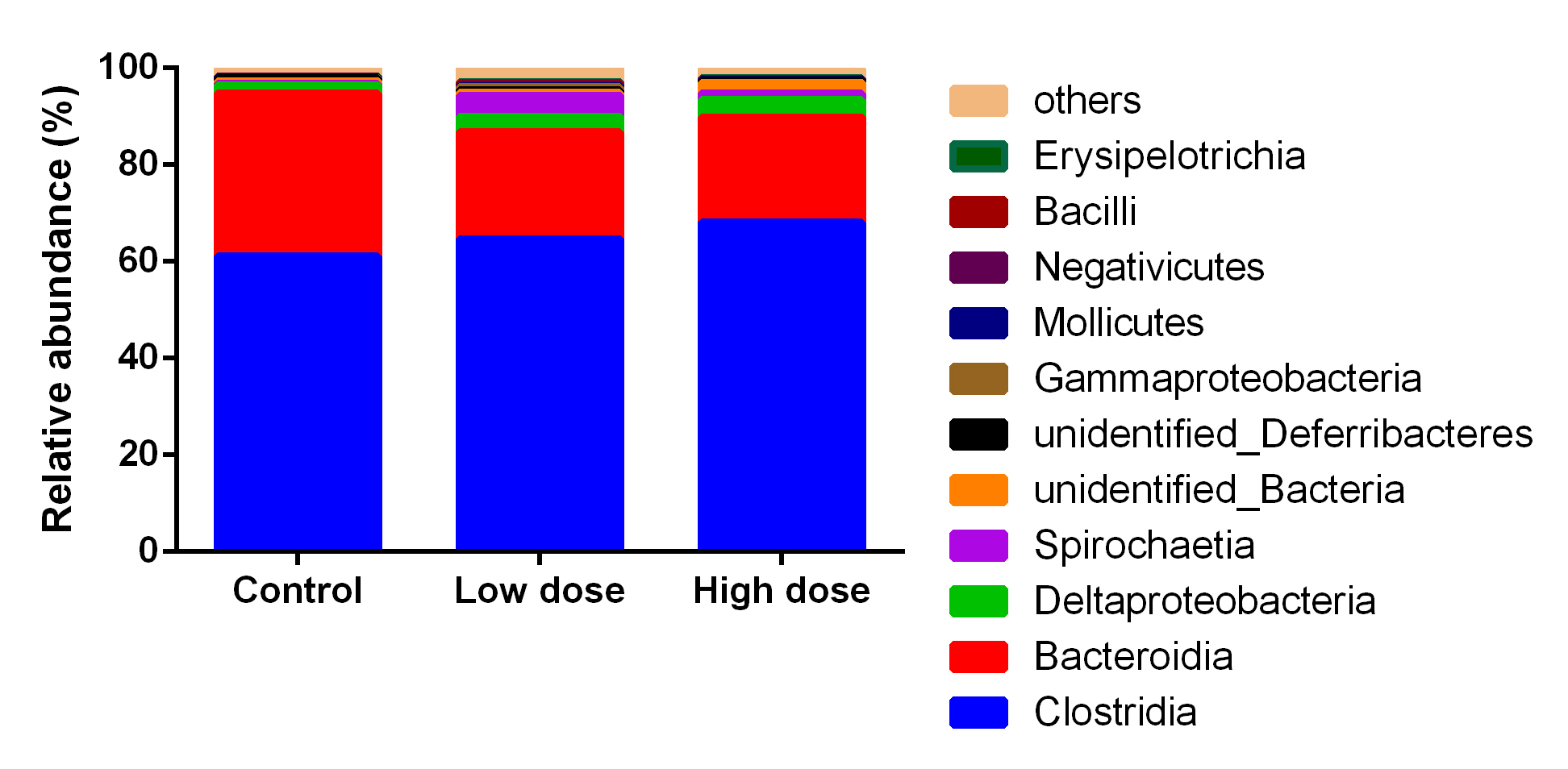

Supplement: Supplementary Figure 1 — Relative abundance of operational taxonomic units among control group (0 mg/kg 6-MBOA), low 6-MBOA dose group (1 mg/kg 6-MBOA) and high 6-MBOA dose group (2 mg/kg 6-MBOA) at the class level in the cecal microbiota of adult male Brandt’s vole. Others mean the classes with relative abundance less than 0.1%. [file Image_1.JPEG]

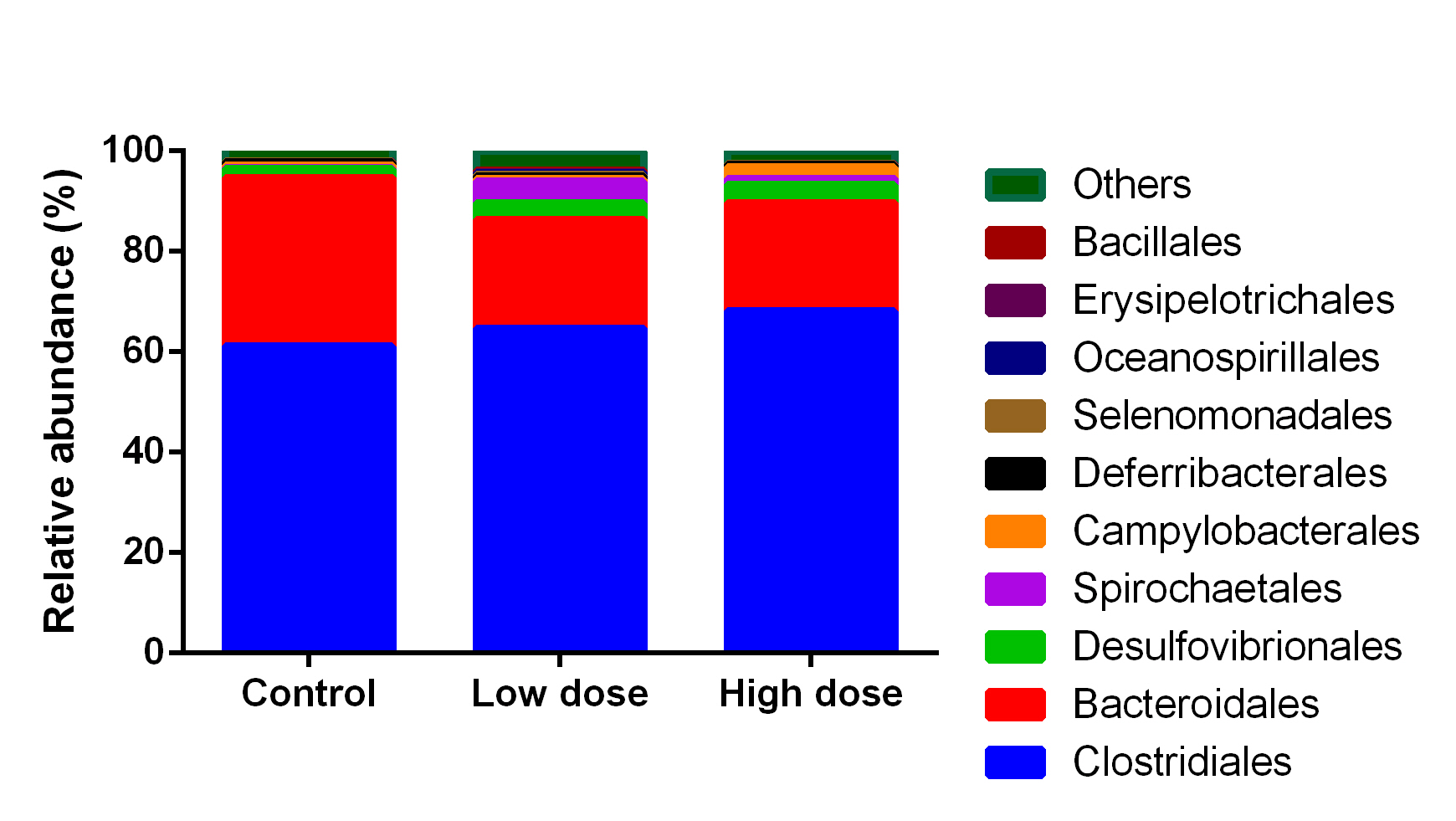

Supplement: Supplementary Figure 2 — Relative abundance of operational taxonomic units among control group (0 mg/kg 6-MBOA), low 6-MBOA dose group (1 mg/kg 6-MBOA) and high 6-MBOA dose group (2 mg/kg 6-MBOA) at the order level in the cecal microbiota of adult male Brandt’s vole. Others mean the orders with relative abundance less than 0.1%. [file Image_2.JPEG]

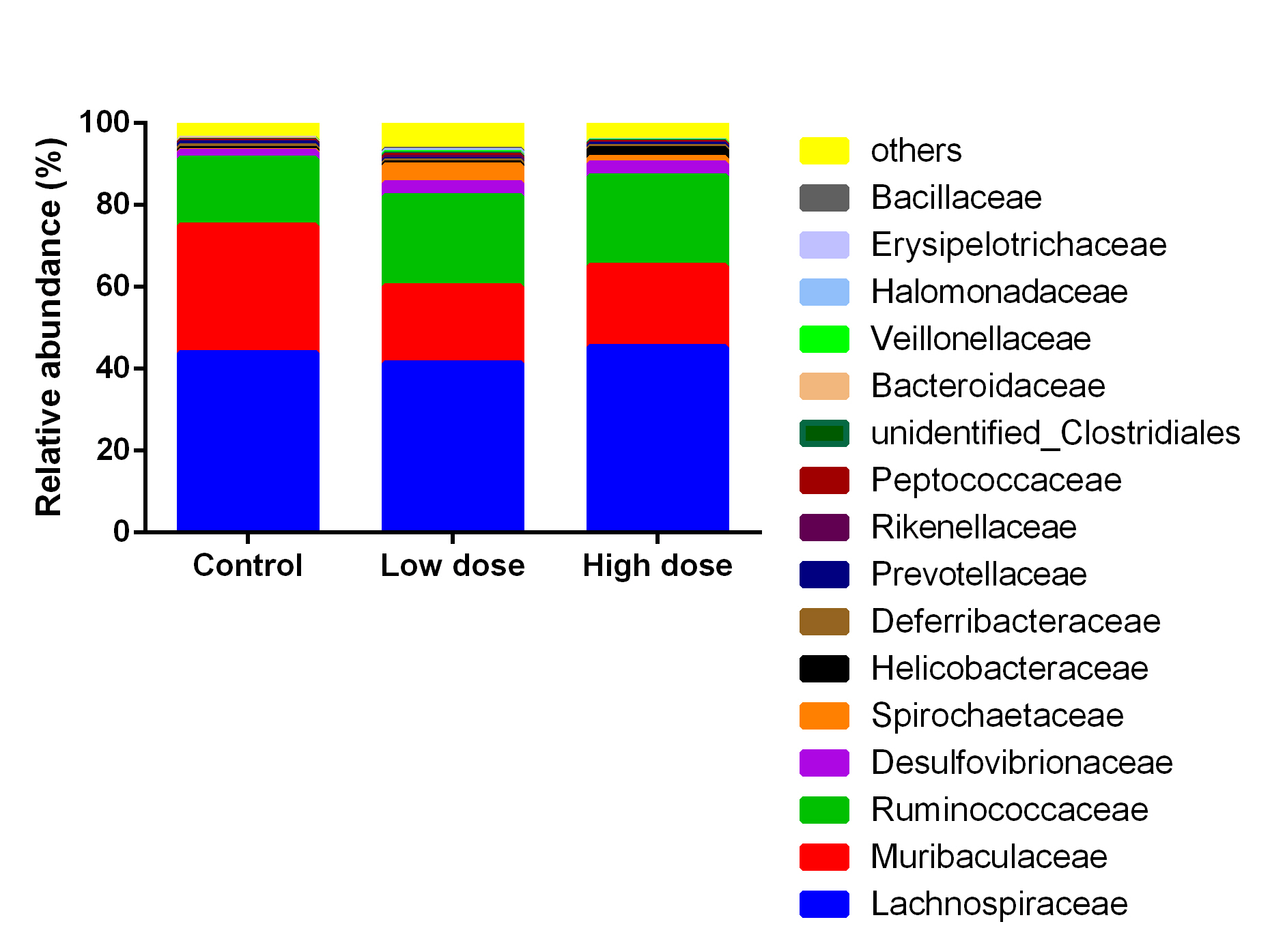

Supplement: Supplementary Figure 3 — Relative abundance of operational taxonomic units among control group (0 mg/kg 6-MBOA), low 6-MBOA dose group (1 mg/kg 6-MBOA) and high 6-MBOA dose group (2 mg/kg 6-MBOA) at the family level in the cecal microbiota of adult male Brandt’s vole. Others mean the families with relative abundance less than 0.1%. [file Image_3.JPEG]

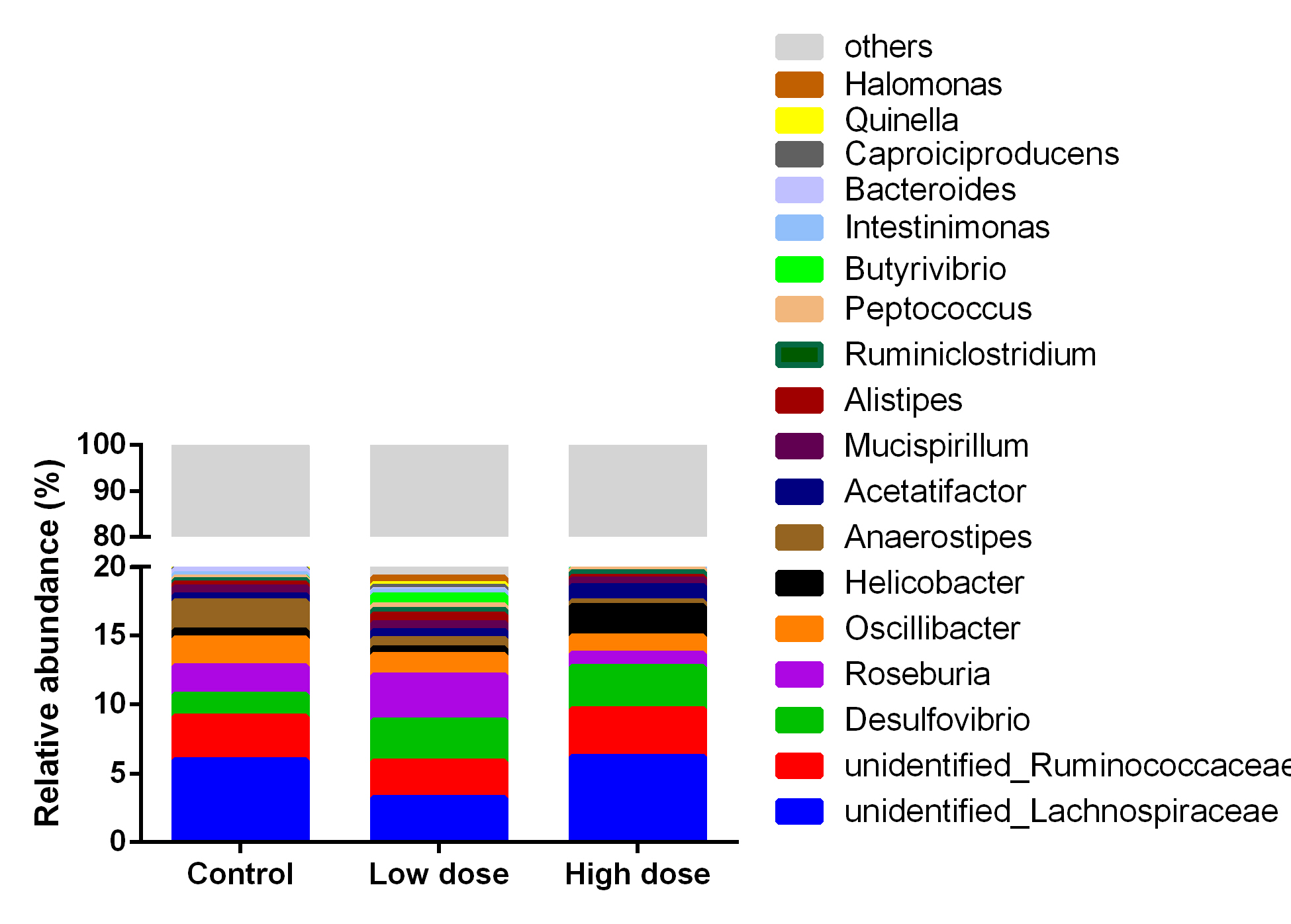

Supplement: Supplementary Figure 4 — Relative abundance of operational taxonomic units among control group (0 mg/kg 6-MBOA), low 6-MBOA dose group (1 mg/kg 6-MBOA) and high 6-MBOA dose group (2 mg/kg 6-MBOA) at the genus level in the cecal microbiota of adult male Brandt’s vole. Others mean the genera with relative abundance less than 0.1%. [file Image_4.JPEG]
